# Influence of heparin-based anticoagulants on antibiotic therapy

**DOI:** 10.3389/fimmu.2025.1708169

**Published:** 2025-11-11

**Authors:** Denisa Cont, Claudia Schildböck, Claudia Kolm, Alexander K. T. Kirschner, Andreas H. Farnleitner, Jens Hartmann, Viktoria Weber, Stephan Harm

**Affiliations:** 1 Department for Biomedical Research, University for Continuing Education Krems, Krems an der Donau, Austria; 2 Department of Physiology, Pharmacology and Microbiology, Division Water Quality and Health, Karl Landsteiner University of Health Sciences, Krems an der Donau, Austria; 3 Institute of Chemical, Environmental and Bioscience Engineering, Research Group Microbiology and Molecular Diagnostics, Vienna University of Technology, Vienna, Austria; 4 Institute for Hygiene and Applied Immunology, Water Microbiology, Medical University of Vienna, Vienna, Austria

**Keywords:** host defense peptides, antibiotics, heparin-based anticoagulants, ESKAPE pathogens, antimicrobial compounds

## Abstract

**Background:**

A world without antibiotics is hard to conceive. They have revolutionized the treatment landscape for bacterial infections, reducing mortality rates and enabling complex medical procedures. However, their widespread use has fueled the rise of antimicrobial resistance, a growing global health threat that demands new antibacterial therapies and strategies to preserve the efficacy of existing treatments. Among promising candidates, antimicrobial compounds (AMCs) offer broad-spectrum antimicrobial activity with a lower risk of resistance development. Recent studies suggest that unfractionated heparin, a commonly used anticoagulant, reduces the antibacterial and endotoxin-neutralizing activity of blood-derived AMCs, likely through ionic interactions.

**Methods:**

Given the prevalence of negatively charged anticoagulants in clinical settings, we aimed to explore the effects of unfractionated heparin, low molecular weight heparin, and fondaparinux on the antibacterial activity of AMCs and antibiotics (colistin, daptomycin, gentamicin, imipenem, ofloxacin, and vancomycin).

**Results:**

Our results revealed that both unfractionated and low molecular weight heparin markedly impaired the antibacterial activity of AMCs and positively charged antibiotics, whereas fondaparinux showed no such effect. For instance, exposure to 2.5 IU/mL of unfractionated and low molecular weight heparin led to a significant increase in the minimal inhibitory and minimal bactericidal concentrations of colistin and gentamicin.

**Conclusions:**

These findings support our hypothesis that specific heparin-based anticoagulants interfere with the activity of blood-derived AMCs and positively charged antibiotics, reducing their efficacy *in vitro*. Our research aims to provide a foundation for future studies focused on optimizing anticoagulant use in clinical settings, ultimately improving patient outcomes in the ongoing fight against multidrug-resistant bacteria.

## 1 Introduction

Antibiotics are critical in the fight against pathogenic bacteria (1). The foundation of antibacterial therapy was laid in 1909 with the synthesis of Salvarsan by Paul Ehrlich and Sahachiro Hata, the first drug specifically designed to target bacterial infections (2, 3). This breakthrough was followed by Alexander Fleming’s discovery of penicillin in 1928, widely regarded as the first true antibiotic (4, 5). By significantly lowering mortality rates and enabling safer medical procedures, antibiotics remain indispensable in healthcare, consistently ranking among the most frequently prescribed medications globally (6).

The effectiveness of antibiotics, however, is increasingly compromised by antimicrobial resistance (AMR), which the World Health Organization (WHO) has declared as one of the most pressing global health challenges of the 21^st^ century. Multidrug-resistant (MDR) bacteria, capable of evading multiple antibiotics, emerged through genetic mutations and horizontal gene transfer, but the misuse and overuse of antibiotics in healthcare and factory farming have exacerbated this phenomenon (7–10). The impact is alarming: in 2019, AMR was directly responsible for 1.27 million deaths, with an additional 4.95 million deaths associated with resistant infections (11). Among the most concerning MDR bacteria are the ESKAPE pathogens—*Enterococcus faecium*, *Staphylococcus aureus*, *Klebsiella pneumoniae*, *Acinetobacter baumannii*, *Pseudomonas aeruginosa*, and *Enterobacter* spp.—which are highly pathogenic, resistant to multiple drugs, and listed by the WHO as priority pathogens in urgent need of new treatment options (12, 13). Untreated or inadequately treated infections can escalate to systemic, life-threatening conditions such as sepsis, a dysregulated host response to infection (14, 15). Globally, sepsis remains a significant health burden, responsible for 14.1 million deaths in 2019 (16).

The impact of AMR extends far beyond the health of individual patients, creating far-reaching challenges for healthcare systems and economies. Resistant infections lead to prolonged hospital stays, increased healthcare costs, and the need for costly second- and third-line treatments (17, 18). To combat AMR and avert a post-antibiotic era, a multifaceted approach is essential, including the discovery of novel antibiotics and improved stewardship to preserve the efficacy of existing therapies (19–21). While new antibiotics are urgently needed, only few new compounds have been developed in recent decades, such as teixobactin (2015) and clovibactin (2023), both discovered using iChip technology (22, 23). Meanwhile, alternative strategies like antimicrobial compounds (AMCs) have gained considerable attention. These naturally occurring, cationic molecules are integral components of the innate immune system, exhibiting potent broad-spectrum antimicrobial and immunomodulatory properties (24, 25). Unlike conventional antibiotics, AMCs act through nonspecific mechanisms, making them less prone to resistance development and positioning them as promising tools against MDR pathogens (26–28).

Recent studies have revealed that blood-derived AMCs can be neutralized by unfractionated heparin (UFH), a negatively charged polysaccharide with anticoagulant and anti-inflammatory properties (29–31). This interaction, potentially resulting from ionic interaction between the anionic UFH and the cationic molecules, has been shown to reduce the antibacterial and endotoxin-neutralizing efficacy of AMCs. Given the use of polyanionic anticoagulants in clinical settings, concerns arise regarding the potential of UFH and related anticoagulants to interfere with AMCs and positively charged antibiotics. Therefore, in the present study, we investigated the impact of various heparin-based anticoagulants on the activity of blood-derived AMCs and antibiotics (colistin, daptomycin, gentamicin, imipenem, ofloxacin, and vancomycin) against pathogenic bacteria *in vitro*.

## 2 Materials and methods

### 2.1 Serum samples, antibiotics, and anticoagulants

Human whole blood was collected from healthy volunteer donors into vacutainer tubes (Vacuette CAT Serum Clot Activator tubes, Greiner Bio-One, Kremsmünster, Austria). After clotting, samples were centrifuged at 3500 x g for 10 min. The resulting serum was aliquoted and stored at -20°C until further use. Blood donations were approved by the Ethics Committee of the University for Continuing Education Krems (EK GZ 13/2015-2018). Experiments were conducted in accordance with the guidelines of the Declaration of Helsinki of the World Medical Association. The participants provided their written informed consent to participate in this study. Clinically relevant antibiotics were selected based on their possession of at least one positively charged side group, which could potentially interact with the negatively charged anticoagulants (**Table 1**).

**Table 1:** Pharmacochemical properties of clinically relevant antibiotics.

Antibiotic	Abbrev.	Class	Negative side groups	Positive side groups	Net charge (pH = 7.4)	Ref.
colistin	COL	polymyxin	0	5	+5	(32)
ofloxacin	OFL	fluoroquinolone	1	1	0	(33)
vancomycin	VAN	glycopeptide	1	2	+1	(34)
daptomycin	DAP	lipopeptide	4	1	-3	(35)
gentamicin	GEN	aminoglycoside	0	5	+5	(36)
imipenem	IMI	carbapenem	1	1	0	(37)

Colistin sulfate (COL), ofloxacin (OFL), and vancomycin hydrochloride (VAN) were acquired from Sigma Aldrich (St. Louis, MO), while daptomycin (DAP), gentamicin sulfate (GEN), and imipenem monohydrate (IMI) were obtained from Santa Cruz Biotechnology (Dallas, TX). The anticoagulants used in this study were unfractionated heparin (UFH, Gilvasan Pharma GmbH, Vienna, Austria), low molecular weight heparin (LMWH, Lovenox, Sanofi, Paris, France), and fondaparinux, a synthetic factor Xa inhibitor (FPX, Arixtra, Viatris, Canonsburg, PA). For this study, the heparin-based anticoagulants were dosed according to their anticoagulant effect, expressed in International Units (IU). Detailed conversion calculations are provided in **Supplementary Materials S1**.

### 2.2 Bacterial strains and culture conditions

Luria-Bertani Broth (LB), Mueller-Hinton Broth (MHB) and Nutrient Agar (NA) were purchased from Carl Roth (Karlsruhe, Germany). Four clinically relevant pathogens were used: *A. baumannii* ATCC 19606, *E. faecium* DSM 20477, *E. coli* ATCC 25299, and *S. aureus* DSM 20232. These strains were preserved in glycerol stocks at -80°C for long-term storage and reactivated by culture on NA plates. Before each experiment, overnight cultures were grown in MHB at 37°C.

### 2.3 Anticoagulant-induced neutralization of blood-derived AMCs

Serum samples from six donors were pre-incubated with 5, 25, and 50 IU/mL UFH, LMWH, and FPX for 4 h at 37°C. A bacterial suspension of *E. coli* was prepared in LB to an optical density of 0.20 ± 0.02 at 600 nm, corresponding to 3x10^8^ colony-forming units (CFU)/mL based on McFarland standards. This suspension was then diluted to a final concentration of 3x10^4^ CFU/mL. Following pre-incubation, the serum samples were mixed in a 1:1 ratio with the bacterial suspension and incubated for 18 h at 37°C. Bacterial growth was assessed indirectly by measuring the absorbance at 600 nm.

### 2.4 Screening the impact of heparin-based anticoagulants on antibiotic activity

The minimal inhibitory concentration (MIC) was determined using the broth microdilution method, following the Clinical and Laboratory Standards Institute (CLSI) guidelines for antimicrobial susceptibility testing (38). COL, DAP, GEN, IMI, OFL, and VAN were prepared at an initial concentration of 5.12 mg/mL in sterile distilled water and serially diluted (64 – 0.06 µg/mL) in a 96-well plate containing cation-adjusted MHB (CAMHB), supplemented with 50 µg/mL calcium for DAP. Pre-incubated serum with 50 IU/mL UFH, LMWH, and FPX was mixed at a 1:1 ratio with each antibiotic dilution for 4 h at 37°C, resulting in a final anticoagulant concentration of 25 IU/mL. Following incubation, samples were spiked with a final bacterial suspension adjusted to 1.5x10^6^ CFU/mL, prepared from a 1.5x10^8^ CFU/mL stock in MHB (0.5 McFarland standard). Controls were conducted using native serum and saline solution. *A. baumannii* and *E. coli* were tested against COL, GEN, IMI, and OFL, while *E. faecium* and *S. aureus* were tested against DAP, GEN, IMI, and VAN. After 18 ± 2 h of incubation at 37°C, MIC values were determined as the lowest concentration of antibiotic that inhibited visible bacterial growth, measured by absorbance at 600 nm.

### 2.5 Evaluation of antibiotic efficacy at lower anticoagulant concentrations

Considering previous results, antibiotics affected by the presence of 25 IU/mL heparin-based anticoagulants were further tested at lower anticoagulant concentrations. Using the same broth microdilution method outlined earlier, each serial dilution of COL and GEN was mixed at a 1:1 ratio with serum from six donors previously pre-incubated with 5 and 25 IU/mL UFH, LMWH, and FPX for 4 h at 37°C, ending with a final anticoagulant concentration of 2.5 and 12.5 IU/mL. Controls were conducted using native serum and saline solution. The samples were spiked with 1.5x10^6^ CFU/ml (final concentration) of *A. baumannii, E. coli, E. faecium* and *S. aureus* in the case of GEN and with *A. baumannii* and *E. coli* in the case of COL. After 18 ± 2 h incubation at 37°C, the MIC and the minimal bactericidal concentration (MBC) were determined, and DNA was quantified using qPCR. For MBC assessment, a volume of 10 µL was removed from wells without visible growth at 600 nm and incubated overnight on NA plates at 37°C. MBC was defined as a >99.9% reduction of the initial colony counts, whereby the threshold value for our experiment was 10 CFUs. The qPCR protocol and sequence of the in-house designed primers used for the bacterial DNA quantification are given in Cont et al., 2024 (31).

### 2.6 Statistical analysis

Experiments were conducted in duplicates. Statistical analyses were performed using R version 4.4.2 (R Foundation for Statistical Computing, Vienna, Austria). Ct values were log-transformed prior to analysis to meet model assumptions. Normality of residuals was verified using the Shapiro–Wilk test. When residuals were normally distributed, differences among conditions were assessed using a repeated-measures mixed-effects model (random intercept for donor) followed by Dunnett’s *post-hoc* test to compare each treatment with native serum. In cases where residuals deviated from normality, non-parametric Friedman tests followed by paired Wilcoxon *post-hoc* tests with Holm adjustment were applied. Significance levels were defined as follows: ns p > 0.05, * p ≤ 0.05, ** p ≤ 0.01, *** p ≤ 0.001, **** p ≤ 0.0001. Data visualization was conducted in GraphPad Prism 9.3.1 (GraphPad Software, Boston, MA). For calculation purposes, MIC and MBC values below 0.06 µg/mL were considered as 0.06 µg/mL to simplify data processing.

## 3 Results

### 3.1 Anticoagulant-induced neutralization of blood-derived AMCs

**Figure 1:**
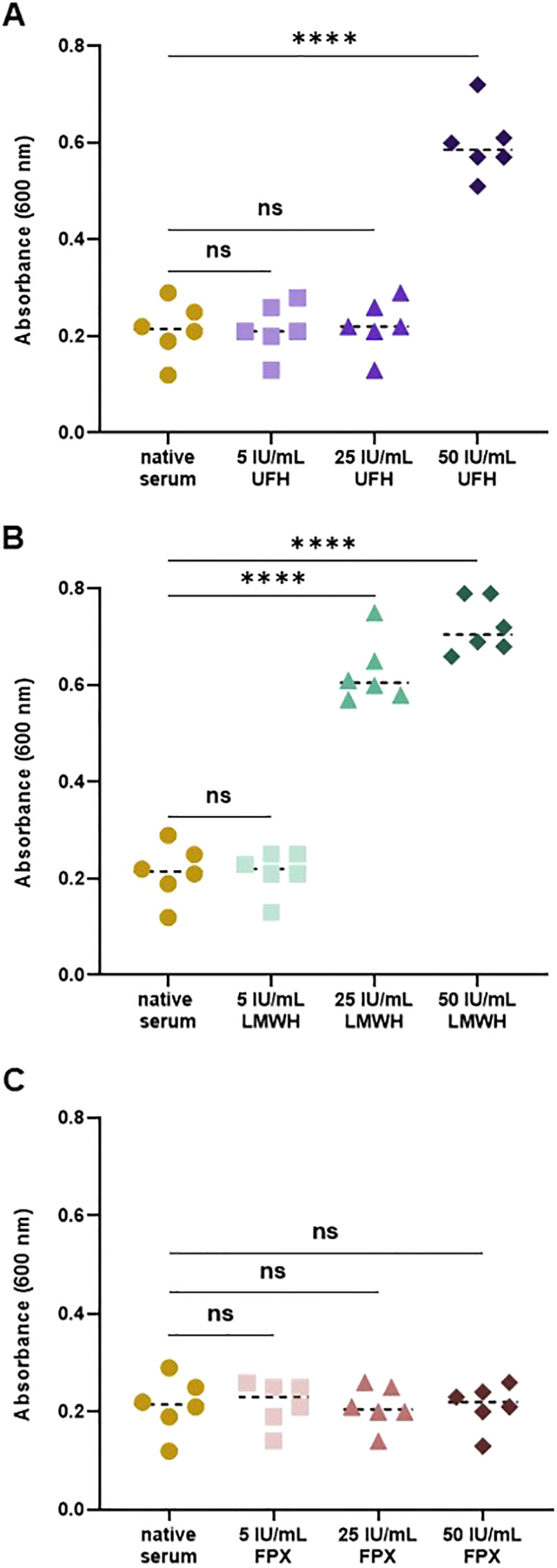
Impact of heparin-based anticoagulants on the antibacterial activity of blood-derived AMCs. Serum samples were pre-incubated with 5, 25, and 50 IU/mL UFH **(A)**, LMWH **(B)**, and FPX **(C)** for 4 h at 37°C. Following pre-incubation, samples were mixed in a 1:1 ratio with an *E*. *coli* suspension (3x10^4^ CFU/mL) and incubated for 18 h at 37°C. Bacterial growth was quantified indirectly by measuring absorbance at 600 nm (n = 6).

When assessing the effects of UFH, LMWH, and FPX on the antibacterial activity of blood-derived AMCs, we observed that both UFH and LMWH significantly enhanced the growth of *E. coli*, as indicated by higher absorbance values compared to native serum (**Figure 1**). This effect was dose-dependent: LMWH induced an increase in absorbance starting at 25 IU/mL, while UFH showed a similar response from 50 IU/mL. In contrast, absorbance values in FPX-spiked serum were similar to those in native serum, suggesting no significant impact on bacterial growth at any concentration tested.

### 3.2 Screening the impact of heparin-based anticoagulants on antibiotic activity

To evaluate the influence of heparin-based anticoagulants on antibiotic efficacy against *A. baumannii*, *E. coli*, *E. faecium*, and *S. aureus*, variations in the MIC values of COL, DAP, GEN, IMI, OFL, and VAN were assessed in the presence of 25 IU/mL of UFH, LMWH, FPX. The results revealed that these anticoagulants notably interfered with the activity of COL and GEN in the gram-negative bacteria tested, while IMI and OFL remain unaffected (**Table 2**). In *A. baumannii*, UFH- and LMWH-spiked serum resulted in a 4.2-fold and 33.3- fold MIC increase for COL and GEN, respectively, whereas FPX-spiked serum had no measurable impact on their MIC. Similarly, in *E. coli*, UFH and LMWH caused a MIC increase of COL and GEN by no less than 4-fold. FPX exhibited a moderate effect, raising the MIC of COL and GEN by 2-fold and 4.2-fold, respectively.

**Table 2:** Impact of 25 IU/mL UFH, LMWH, and FPX on the efficacy of COL, GEN, IMI, and OFL against *A. baumannii* and *E. coli*.

			COL		GEN		IMI		OFL	
			MIC (µg/mL)	MIC fold change	MIC (µg/mL)	MIC fold change	MIC (µg/mL)	MIC fold change	MIC (µg/mL)	MIC fold change
*A. baumannii*	Saline	Un-spiked saline	4		1		0.5		0.5	
		UFH-saline	8	2.0	**4**	**4.0**	0.5	1.0	0.5	1.0
		LMWH-saline	8	2.0	**4**	**4.0**	0.5	1.0	0.5	1.0
		FPX-saline	4	1.0	2	**2.0**	0.5	1.0	0.5	1.0
	Serum	Native serum	<0.06		<0.06		<0.06		<0.06	
		UFH-serum	0.25	4.2	2	**33.3**	<0.06	1.0	<0.06	1.0
		LMWH-serum	0.25	4.2	2	**33.3**	<0.06	1.0	<0.06	1.0
		FPX-serum	<0.06	1.0	<0.06	1.0	<0.06	1.0	<0.06	1.0
*E. coli*	Saline	Un-spiked saline	4		0.25		2		>0.06	
		UFH-saline	8	2.0	1	**4.0**	2	1.0	>0.06	1.0
		LMWH-saline	16	4.0	1	**4.0**	2	1.0	>0.06	1.0
		FPX-saline	4	1.0	0.5	**2.0**	2	1.0	>0.06	1.0
	Serum	Native serum	0.25		>0.06		2		>0.06	
		UFH-serum	1	4.0	0.25	**4.2**	2	1.0	>0.06	1.0
		LMWH-serum	2	8.0	0.25	**4.2**	2	1.0	>0.06	1.0
		FPX-serum	0.5	2.0	0.25	**4.2**	2	1.0	>0.06	1.0

Serum samples were pre-incubated for 4 h at 37°C with 50 IU/mL UFH, LMWH, and FPX and then mixed at a 1:1 ratio with serially diluted antibiotics (COL, GEN, IMI, and OFL) from 64 to 0.06 µg/mL. Saline solution served as control. Samples were then spiked with a 1.5x10^6^ CFU/mL bacterial suspension (final concentration) and incubated for 18 ± 2 h at 37°C (n = 3). Bold values indicate an increase in MIC or MBC compared to native serum (control).

Among the gram-positive bacteria, GEN was the only antibiotic affected, while DAP, IMI, and VAN retained their efficacy in the presence of the anticoagulants (**Table 3**). Specifically, serum incubation with 25 IU/mL UFH, LMWH, and FPX resulted in a 4-fold increase in the MIC of GEN against both *E. faecium* and *S. aureus*.

**Table 3:** Impact of 25 IU/mL UFH, LMWH, and FPX on the efficacy of DAP, GEN, IMI, and VAN against *E. faecium* and *S. aureus*.

			DAP		GEN		IMI		VAN	
			MIC (µg/mL)	MIC fold change	MIC (µg/mL)	MIC fold change	MIC (µg/mL)	MIC fold change	MIC (µg/mL)	MIC fold change
*E. faecium*	Saline	Un-spiked saline	2		2		16		1	
		UFH-saline	2	1.0	4	**2.0**	16	1.0	1	1.0
		LMWH-saline	2	1.0	4	**2.0**	16	1.0	1	1.0
		FPX-saline	2	1.0	2	1.0	16	1.0	1	1.0
	Serum	Native serum	2		2		16		1	
		UFH-serum	2	1.0	8	**4.0**	16	1.0	1	1.0
		LMWH-serum	2	1.0	8	**4.0**	16	1.0	1	1.0
		FPX-serum	2	1.0	8	**4.0**	16	1.0	1	1.0
*S. aureus*	Saline	Un-spiked saline	1		0.5		0.5		1	
		UFH-saline	1	1.0	1	**2.0**	0.5	1.0	1	1.0
		LMWH-saline	1	1.0	1	**2.0**	0.5	1.0	1	1.0
		FPX-saline	1	1.0	0.5	1.0	0.5	1.0	1	1.0
	Serum	Native serum	1		0.25		0.5		1	
		UFH-serum	1	1.0	1	**4.0**	0.5	1.0	1	1.0
		LMWH-serum	1	1.0	1	**4.0**	0.5	1.0	1	1.0
		FPX-serum	1	1.0	1	**4.0**	0.5	1.0	1	1.0

Serum samples were pre-incubated for 4 h at 37°C with 50 IU/mL UFH, LMWH, and FPX and then mixed at a 1:1 ratio with serially diluted antibiotics (DAP, GEN, IMI, and VAN) from 64 to 0.06 µg/mL. Saline solution served as control. Samples were then spiked with a 1.5x10^6^ CFU/mL bacterial suspension (final concentration) and incubated for 18 ± 2 h at 37°C (n = 3). Bold values indicate an increase in MIC or MBC compared to native serum (control).

### 3.3 Evaluation of antibiotic efficacy at lower anticoagulant concentrations

To further examine the interaction between heparin-based anticoagulants and antibiotics, those antibiotics susceptible to 25 IU/mL UFH, LMWH, and/or FPX were tested at 2.5 and 12.5 IU/mL anticoagulant concentrations. For COL, 12.5 IU/mL UFH and LMWH resulted in a 4.2-fold and 3.1-fold increase in MIC/MBC against *A. baumannii* and *E. coli*, respectively, compared to native serum (**Table 4**). In contrast, 12.5 IU/mL FPX caused only a slight rise in MIC/MBC against *E. coli* (~1.5-fold) with no notable effect on *A. baumannii*. 2.5 IU/mL UFH and LMWH induced a 2.6-fold increase in MIC/MBC against *A. baumannii*, with a less pronounced effect observed for *E. coli*. At 2.5 IU/mL, FPX did not modify the MIC or MBC in any of the gram-negative bacteria tested.

**Table 4:** Changes in the MIC and MBC values of COL against *A. baumannii* and *E. coli* in the presence of 2.5 and 12.5 IU/mL UFH, LMWH, and FPX.

		2.5 IU/mL anticoagulant				12.5 IU/mL anticoagulant			
		MIC (µg/mL)	MIC fold change	MBC (µg/mL)	MBC fold change	MIC (µg/mL)	MIC fold change	MBC (µg/mL)	MBC fold change
*A. baumannii*	Native serum	<0.06 ± 0.00		<0.06 ± 0.00		<0.06 ± 0.00		<0.06 ± 0.00	
	UFH-serum	0.16 ± 0.09	**2.6**	0.16 ± 0.09	**2.6**	0.25 ± 0.00	**4.2**	0.25 ± 0.00	**4.2**
	LMWH-serum	0.16 ± 0.09	**2.6**	0.16 ± 0.09	**2.6**	0.25 ± 0.00	**4.2**	0.25 ± 0.00	**4.2**
	FPX-serum	<0.06 ± 0.00	1.0	<0.06 ± 0.00	1.0	<0.06 ± 0.00	1.0	<0.06 ± 0.00	1.0
*E. coli*	Native serum	0.19 ± 0.06		0.38 ± 0.13		0.19 ± 0.06		0.38 ± 0.13	
	UFH-serum	0.29 ± 0.15	**1.5**	0.42 ± 0.12	**1.1**	0.58 ± 0.19	**3.1**	1.17 ± 0.37	**3.1**
	LMWH-serum	0.27 ± 0.11	**1.4**	0.54 ± 0.22	**1.4**	0.58 ± 0.19	**3.1**	1.17 ± 0.37	**3.1**
	FPX-serum	0.19 ± 0.06	1.0	0.38 ± 0.13	1.0	0.29 ± 0.15	**1.5**	0.58 ± 0.19	**1.6**

Each serial dilution of COL (64 to 0.06 µg/mL) was mixed at a 1:1 ratio with serum previously pre-incubated with 5 and 25 IU/mL UFH, LMWH, and FPX for 4 h at 37°C. Samples were spiked with 1.5x10^6^ CFU/mL (final concentration) of *A. baumannii* and *E. coli*. After 18 ± 2 h incubation at 37°C, results were compared trough MIC and MBC (mean ± SD; n = 6). Bold values indicate an increase in MIC or MBC compared to native serum (control).

In case of GEN, 2.5 and 12.5 IU/mL UFH resulted in an 11.3-fold and 20.8-fold increase, respectively, in the MIC/MBC for *A. baumannii*, with similar effects observed in LMWH-spiked serum (**Table 5**). In contrast, no impact on GEN activity against *A. baumannii* was noted in the presence of FPX at any concentration tested. For *E. faecium* and *S. aureus*, a similar effect on GEN activity was observed, where 2.5 IU/mL UFH and LMWH caused a ~2-fold rise in the MIC/MBC values. Conversely, FPX at 2.5 IU/mL did not interfere with GEN efficacy in these species. In *E. coli*, all drugs tested had a mild impact on GEN activity, with MIC/MBC values exhibiting a roughly 2-fold change in the presence of UFH and LMWH, and a modest 1.2-fold alteration with FPX. MIC values from the controls performed in saline solution can be found in **Supplementary Materials S2**.

**Table 5:** Changes in the MIC and MBC values of GEN against *A. baumannii*, *E. coli*, *E. faecium*, and *S. aureus* in the presence of 2.5 and 12.5 IU/mL UFH, LMWH, and FPX.

		2.5 IU/mL anticoagulant				12.5 IU/mL anticoagulant			
		MIC (µg/mL)	MIC fold change	MBC (µg/mL)	MBC fold change	MIC (µg/mL)	MIC fold change	MBC (µg/mL)	MBC fold change
*A. baumannii*	Native serum	<0.06 ± 0.00		<0.06 ± 0.00		<0.06 ± 0.00		<0.06 ± 0.00	
	UFH-serum	0.68 ± 0.36	**11.3**	0.68 ± 0.36	**11.3**	1.25 ± 0.56	**20.8**	1.25 ± 0.56	**20.8**
	LMWH-serum	0.77 ± 0.67	**12.8**	0.77 ± 0.67	**12.8**	1.25 ± 0.56	**20.8**	1.25 ± 0.56	**20.8**
	FPX-serum	<0.06 ± 0.00	1.0	<0.06 ± 0.00	1.0	<0.06 ± 0.00	1.0	<0.06 ± 0.00	1.0
*E. coli*	Native serum	0.21 ± 0.06		0.42 ± 0.12		0.21 ± 0.06		0.42 ± 0.12	
	UFH-serum	0.38 ± 0.13	**1.8**	0.75 ± 0.25	**1.8**	0.46 ± 0.09	**2.2**	0.92 ± 0.19	**2.2**
	LMWH-serum	0.46 ± 0.09	**2.2**	0.92 ± 0.19	**2.2**	0.46 ± 0.09	**2.2**	0.92 ± 0.19	**2.2**
	FPX-serum	0.25 ± 0.00	**1.2**	0.50 ± 0.00	**1.2**	0.38 ± 0.13	**1.8**	0.75 ± 0.25	**1.8**
*E. faecium*	Native serum	2.00 ± 0.00		2.00 ± 0.00		2.00 ± 0.00		2.00 ± 0.00	
	UFH-serum	3.33 ± 0.94	**1.7**	3.33 ± 0.94	**1.7**	5.33 ± 1.89	**2.7**	5.33 ± 1.89	**2.7**
	LMWH-serum	3.33 ± 0.94	**1.7**	3.33 ± 0.94	**1.7**	5.33 ± 1.89	**2.7**	5.33 ± 1.89	**2.7**
	FPX-serum	2.00 ± 0.00	1.0	2.00 ± 0.00	1.0	2.67 ± 0.94	**1.3**	2.67 ± 0.94	**1.3**
*S. aureus*	Native serum	0.33 ± 0.12		0.67 ± 0.24		0.33 ± 0.12		0.67 ± 0.24	
	UFH-serum	0.67 ± 0.24	**2.0**	1.33 ± 0.47	**2.0**	1.00 ± 0.00	**3.0**	2.00 ± 0.00	**3.0**
	LMWH-serum	0.67 ± 0.24	**2.0**	1.33 ± 0.47	**2.0**	1.00 ± 0.00	**3.0**	2.00 ± 0.00	**3.0**
	FPX-serum	0.33 ± 0.12	1.0	0.67 ± 0.24	1.0	0.50 ± 0.00	**1.5**	1.00 ± 0.00	**1.5**

Each serial dilution of GEN (64 to 0.06 µg/mL) was mixed at a 1:1 ratio with serum previously pre-incubated with 5 and 25 IU/mL UFH, LMWH, and FPX for 4 h at 37°C. Samples were spiked with 1.5x10^6^ CFU/mL (final concentration) of *A. baumannii*, *E. coli*, *E. faecium*, and *S.* aureus. After 18 ± 2 h incubation at 37°C, results were compared trough MIC and MBC (mean ± SD; n = 6). Bold values indicate an increase in MIC or MBC compared to native serum (control).

**Figure 2:**
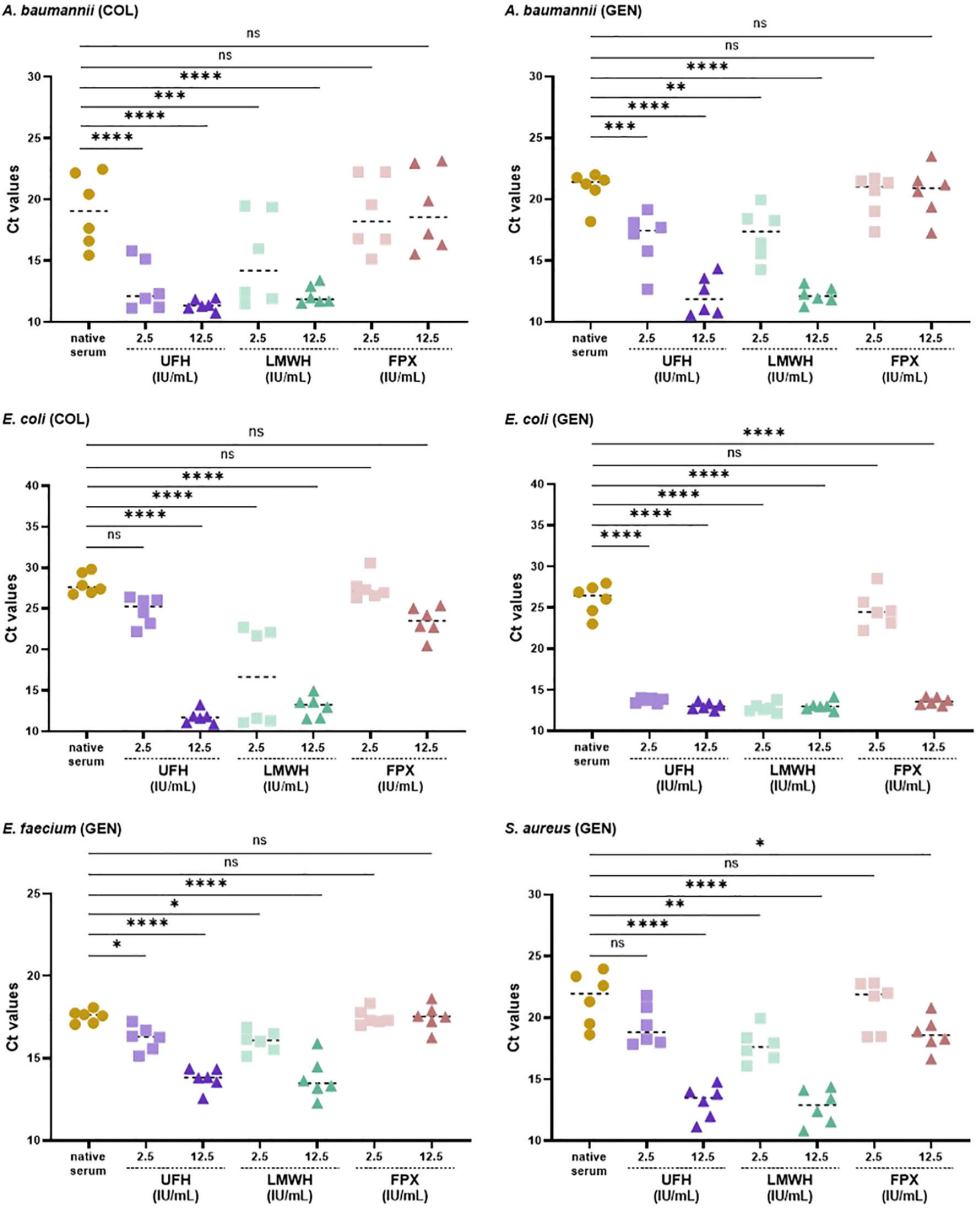
Bacterial load (Ct values) in native and anticoagulant-treated serum (5 or 25 IU/mL UFH, LMWH, FPX) mixed 1:1 with the MIC concentrations of COL and GEN (final anticoagulant concentration of 2.5 and 12.5 IU/mL). The bacterial load was quantified using qPCR as detailed in the Materials and Methods section (n = 6).

Comparing the bacterial load at the effective MIC concentrations of COL and GEN in native serum to serum pre-incubated with 5 and 25 IU/mL UFH, LMWH, and FPX, yielded consistent results (**Figure 2**). 2.5 IU/mL UFH and LMWH resulted in a significant increased the bacterial load of *A. baumannii*, *E. coli*, *E. faecium*, and *S. aureus* for both antibiotics. For instance, when comparing the Ct values between native serum and 2.5 IU/mL UFH and LMWH in combination with COL against *A. baumannii*, we observed a decrease of 6.2 and 4.0 cycles, respectively, correlating with higher bacterial concentration, whereas 2.5 IU/mL FPX showed same values as the native serum.

## 4 Discussion

The introduction of the term “antibiotic” by Selman Waksman in 1942 marked the beginning of the golden age of antibiotics, leading to the development of over 20 classes that revolutionized the treatment of bacterial infections (1, 39–43). Despite these advancements, the discovery of new antibiotics has slowed dramatically in recent years, while AMR continues to rise, emphasizing the urgent need for novel antibacterial therapies and strategies to preserve the efficacy of existing treatments (44). In our previous work, we demonstrated that UFH interferes with the antibacterial and endotoxin-neutralizing activity of blood-derived AMCs, likely due to ionic interactions (31). In this study, we further validated our hypothesis that various heparin-based anticoagulants can neutralize not only AMCs, but also positively charged antibiotics, reducing their efficacy.

Our findings revealed that both UFH and LMWH significantly impaired the antibacterial activity of AMCs, as evidenced by increased absorbance values of *E. coli* compared to native serum, with a reduction in their activity starting at 25 IU/mL for LMWH and 50 IU/mL for UFH, indicating a dose-dependent effect. In contrast, FPX-spiked serum had no effect on AMC efficacy under our experimental conditions. These observations align with our prior findings, which demonstrated that UFH and LMWH interfered with the endotoxin-neutralizing function of blood-derived endotoxin-neutralizing compounds (ENCs, a subclass of AMCs), resulting in increased endotoxin activity, while FPX had no such effect (30). Regarding the dose-dependent effect, we previously showed that a 4-hour pre-incubation with high UFH (50–250 IU/mL) was sufficient to impair AMC activity, whereas lower concentrations, such as 5 IU/mL, required extended exposure times to achieve similar effects (31).

To explore the impact of heparin-based anticoagulants on antibiotic efficacy, we assessed the change in the MIC of COL, DAP, GEN, IMI, OFL, and VAN in the presence of 25 IU/mL UFH, LMWH, and FPX against *A. baumannii*, *E. coli*, *E. faecium*, and *S. aureus*. Our data provide evidence that these heparin-based anticoagulants notably diminished the activity of COL and GEN, while DAP, IMI, OFL, and VAN remained unaffected. Specifically, pre-incubation with 25 IU/mL of UFH, LMWH, and FPX resulted in MIC increases of over 4-fold for COL and GEN across all pathogens, compared to native serum, except in FPX-serum tested with COL against *E. coli*, which showed only a 2-fold rise. As anticipated, these anionic anticoagulants only affected positively charged antibiotics (COL and GEN), with no impact observed on antibiotics with either a weakly positive (VAN), neutral (IMI, OFL), or negative (DAP) net charge (see **Table 1**). These findings suggest that the negatively charged polysaccharide backbone of UFH and LMWH likely interacts electrostatically with cationic antibiotics such as COL and GEN, reducing their free active fraction and consequently limiting their bactericidal efficacy.

Higher anticoagulant concentrations were initially used to screen for potential interference with antibiotic activity, and for those conditions where an effect was observed, lower concentrations were subsequently tested to confirm the physiological relevance of the findings. When testing lower anticoagulant concentrations, 2.5 IU/mL UFH or LMWH were sufficient to impair the activity of COL and GEN across all bacterial strains tested. Specifically, 2.5 IU/mL of UFH and LMWH led to an approximately 2-fold increase in the MIC and MBC for COL and GEN, whereas 2.5 IU/mL of FPX did not alter the efficacy of any of these antibiotics. Our results reinforce the hypothesis that ionic interactions are likely the primary mechanism behind the neutralizing effects observed. The differences between the heparin-based anticoagulants stem from the varying dosages needed to achieve an equivalent anticoagulant effect. Notably, a much lower dose of FPX is required to have an anticoagulant effect comparable to that of UFH or LMWH. According to the conversion analyses conducted (see **Supplementary Material S1**), a dose of 1.41 µg/mL of FPX is sufficient to attain the anticoagulant effect of 1 IU/mL in human whole blood, whereas 6.34 µg/mL UFH and 9.66 µg/mL LMWH are required.

FXP has been demonstrated to be superior to UFH and LMWH in terms of achieving a comparable anticoagulant effect while minimizing the impact on the antibacterial activity of blood-derived AMCs and cationic antibiotics (COL, GEN). In contrast to our findings, Szekeres et al. (45) reported that heparin enhances the bactericidal activity of COL. However, their use of *E. coli* K12, a non-pathogenic laboratory strain, along with experiments performed in LB instead of blood-derived samples, may account for this discrepancy.

Although we tested the anticoagulants against COL and GEN under identical experimental conditions, the results varied slightly depending on the bacterial strain. As reported in our previous work, bacterial strains exhibit different susceptibilities to blood-derived AMCs and therefore require different serum levels of UFH to affect their growth (31). Interestingly, in *A. baumannii* and *E. coli*, the MIC values for all antibiotics tested were two to four times lower in serum compared to controls performed in saline solution. This suggests a synergistic effect of the blood-derived AMCs and the antibiotics in the Gram-negative bacteria tested. Inter-donor differences represent another factor contributing to the variability in our results, likely arising from variations in AMC levels and susceptibility to heparin-based anticoagulants among individuals. Our previous findings showed a higher endotoxin-neutralizing activity for female serum compared to male serum (46). Moreover, females often have better outcomes in sepsis, including lower in-hospital mortality rates and an improved response to traumatic injury (47). Further research into whether differences in the synthesis and baseline concentrations of blood-derived AMCs, as well as the susceptibility to anionic anticoagulants, are influenced by factors such as age, gender, and overall health status could thus provide valuable insights into why some individuals are more prone to developing sepsis than others. Additionally, considering our previous observation that UFH and LMWH interfere with the endotoxin-neutralizing activity of blood-derived AMCs, and given that COL and GEN are cationic antibiotics with inherent endotoxin-neutralizing capacity, it is plausible that these anticoagulants could similarly impair the endotoxin-neutralizing effect of these antibiotics through electrostatic competition.

While anticoagulants are commonly used as adjunctive therapies to prevent disseminated intravascular coagulation (DIC) in septic patients, it remains inconclusive whether UFH and LMWH are the most effective options. Several clinical studies and meta-analyses have suggested that UFH and LMWH may be associated with decreased mortality (48–51); however, their overall impact remains uncertain (52–55). A recent meta-analysis from 2024, involving 426 septic patients treated with UFH or LMWH, found no significant difference in 28-day and in-hospital mortality between the heparin and control groups, indicating the need for further investigation into the efficacy and safety of this anticoagulants in sepsis (56). Additionally, understanding the interaction between blood-derived AMCs and heparin-based anticoagulants is not only crucial in the context of sepsis but also has broader implications, for example for patients undergoing extracorporeal blood purification, where UFH and LMWH remain the most frequently used anticoagulants (57, 58).

Given that UFH and LMWH strongly bind to AMCs and positively charged antibiotics, it is reasonable to propose that heparan sulfate, a key glycosaminoglycan of the endothelial glycocalyx, may interact with them in a similar manner. The endothelial glycocalyx is a dynamic, negatively charged layer on the luminal surface of vascular endothelial cells, crucial for a variety of physiological and pathological processes (59, 60). Dysfunction and degradation of the glycocalyx, hallmark features of sepsis, compromise vascular integrity, disrupt cell signaling, and amplify inflammation, all of which contribute to disease progression (61, 62). We hypothesize that the glycocalyx may regulate AMC concentrations to prevent cytotoxic effects and may also serve as a reservoir for their release when needed. The AMC-glycocalyx interaction may represent an unrecognized innate immune strategy, forming a protective barrier on the vascular surface to block pathogen entry and mitigate systemic inflammation during localized infections. These insights could also guide the design of medical surfaces with dual functions: promoting blood compatibility while enabling self-coating with AMCs to reduce infection risks and biofilm formation on blood-contacting devices.

To conclude, the complex interplay between heparin-based anticoagulants, antibiotics, glycocalyx, and blood-derived AMCs involves dynamics that warrant deeper investigation. Such interactions may impair bacterial clearance, lead to treatment failure, prolong infections, and contribute to the development of AMR. This research lays the foundation for future studies aimed at optimizing anticoagulant use in clinical settings, minimizing interference with antibiotics and AMCs, and ultimately improving patient outcomes while addressing the spread of MDR bacteria.

## Data Availability

The original contributions presented in the study are included in the article/**Supplementary Material**. Further inquiries can be directed to the corresponding author.
