# Supplementary material for: Influence of heparin-based anticoagulants on antibiotic therapy
Source: Front Immunol. 2025 Nov 11;16:1708169. doi: 10.3389/fimmu.2025.1708169 (PMC12643974; doi:10.3389/fimmu.2025.1708169)
Supplement: Supplementary file 1 [file DataSheet1.docx]

Supplementary Material

1. **Supplementary Figure**

|  |  |
| --- | --- |
|  | 1 IU/mL UFH = 6.34 µg/mL  1 IU/mL LMWH = 9.66 µg/mL  1 IU/mL FPX = 1.41 µg/mL |

**Supplementary Materials S1.** Detailed conversion of anticoagulant concentration to anticoagulant activity. To determine the concentration (µg/mL) required to achieve an anticoagulant activity of 1 IU/mL, plasma samples were spiked with unfractionated heparin (UFH), low-molecular weight heparin (LMWH), and fondaparinux (FPX) at concentrations ranging from 0.5 to 10 µg/mL. Anticoagulant’s activity was measured using the Sysmex CA660 device (Sysmex, Kobe, Japan) and the INNOVANCE® Heparin reagent (Siemens Healthineers, Vienna, Austria). This automated chromogenic assay quantitatively determines the activity of heparin in human citrated plasma. The assay operates by introducing a chromogenic substrate and factor Xa into the plasma sample. The heparin-antithrombin III complex present in the plasma inhibits factor Xa, and the remaining factor Xa converts the chromogenic substrate. The extent of inhibition, which is inversely proportional to the heparin activity in the sample, serves as an indirect measure of the anticoagulant's activity [IU/mL].

1. **Supplementary Table**

|  | *A. baumannii* | | *E. coli* | | *E. faecium* | *S. aureus* |
| --- | --- | --- | --- | --- | --- | --- |
|  | **COL** | **GEN** | **COL** | **GEN** | **GEN** | **GEN** |
| *Un-spiked saline* | 2 | 1 | 2 | 0.5 | 0.5 | 0.25 |
| *UFH-saline* |  |  |  |  |  |  |
| 2.5 IU/mL | 4 | 2 | 4 | 1 | 0.5 | 1 |
| 12.5 IU/mL | 8 | 2 |  | 2 | 2 | 1 |
| *LMWH-saline* |  |  |  |  |  |  |
| 2.5 IU/mL | 4 | 2 | 4 | 1 | 1 | 1 |
| 12.5 IU/mL | 8 | 2 | 4 | 2 | 2 | 1 |
| *FPX-saline* |  |  |  |  |  |  |
| 2.5 IU/mL | 2 | 1 | 2 | 0.5 | 0.5 | 0.25 |
| 12.5 IU/mL | 2 | 1 | 2 | 1 | 1 | 0.5 |

## Supplementary Materials S2. Evaluation of colistin (COL) and gentamicin (GEN) efficacy at 2.5 and 12.5 IU/mL unfractionated heparin (UFH), low molecular weight heparin (LMWH), and fondaparinux (FPX) in saline solution. Each serial dilution of COL and GEN (64 to 0.06 µg/mL) was mixed at a 1:1 ratio with saline solution previously pre-incubated with 5 and 25 IU/mL UFH, LMWH, and FPX for 4 h at 37 °C. Samples were spiked with 1.5x10^6^ CFU/mL of *A. baumannii* and *E. coli* in case of COL and GEN, and with *E. faecium* and *S. aureus* in GEN. After 18 ± 2 h incubation at 37 °C, minimal inhibitory concentration (MIC) values were determined as the lowest concentration of antibiotic that inhibited visible bacterial growth, measured by absorbance at 600 nm.
